# The Role of Cytoreductive Nephrectomy in Renal Cell Carcinoma with Sarcomatoid Histology: A Case Series and Review of the Literature

**DOI:** 10.3390/curroncol29080433

**Published:** 2022-08-03

**Authors:** Hana Studentova, Nikol Rusarova, Andrea Ondruskova, Anezka Zemankova, Vladimir Student, Daniela Skanderova, Bohuslav Melichar

**Affiliations:** 1 Department of Oncology, Faculty of Medicine and Dentistry, University Hospital Olomouc, Palacky University, 771 47 Olomouc, Czech Republic; nikol.rusarova@fnol.cz (N.R.); andrea.ondruskova@fnol.cz (A.O.); anezka.zemankova@fnol.cz (A.Z.); bohuslav.melichar@fnol.cz (B.M.); 2 Department of Urology, Faculty of Medicine and Dentistry, University Hospital Olomouc, Palacky University, 771 47 Olomouc, Czech Republic; vladastudent@gmail.com; 3 Department of Clinical and Molecular Pathology, Faculty of Medicine and Dentistry, University Hospital Olomouc, Palacky University, 771 47 Olomouc, Czech Republic; daniela.skanderova@fnol.cz; 4 Institute of Molecular and Translational Medicine, Faculty of Medicine and Dentistry, University Hospital Olomouc, Palacky University, 771 47 Olomouc, Czech Republic

**Keywords:** cytoreductive nephrectomy, renal cell carcinoma, sarcomatoid, immunotherapy, checkpoint inhibitors

## Abstract

Background: Renal cell carcinoma with sarcomatoid dedifferentiation represents a rare histological entity characterized by aggressive behavior, limited efficacy of tyrosine kinase inhibitors or mTOR inhibitors, and poor outcome. The immune checkpoint inhibitor therapy regimen combining ipilimumab with nivolumab represents a new standard of care for this patient population due to a hitherto unprecedented response rate and overall survival. On the other hand, the role of cytoreductive nephrectomy in metastatic renal cell carcinoma, in particular, with sarcomatoid histology, remains controversial. Patient and Methods: In the present case series, we report six patients with locally advanced or synchronous metastatic sarcomatoid renal cell carcinoma and intermediate or poor International Metastatic RCC Database Consortium (IMDC) risk score, five of whom were successfully subjected to cytoreductive nephrectomy. Results: All six patients received the combination regimen of ipilimumab with nivolumab. Five of these patients underwent upfront cytoreductive nephrectomy followed by systemic treatment without any significant delay, with a durable treatment outcome. Notably, two patients with poor prognostic features achieved a long-term major partial response to therapy. We also performed a review of the literature on optimal treatment strategies for patients with sarcomatoid renal cell carcinoma. Conclusion: Herein, we highlight the feasibility of performing cytoreductive nephrectomy in patients with intermediate/poor prognosis metastatic renal cell carcinoma with sarcomatoid dedifferentiation followed by immunotherapy with ipilimumab and nivolumab. To enhance the chances of immunotherapy success, cytoreductive nephrectomy should also be considered for patients presenting with a disease with adverse prognostic parameters.

## 1. Introduction

Renal carcinoma (RCC) was diagnosed in 431,288 people and contributed to 179,358 deaths worldwide in 2020, according to the GLOBOCAN report [1]. Sarcomatoid differentiation is a relatively unusual feature encountered in about 4–5% of all cases across most histological RCC subtypes, although it is most commonly observed in clear cell RCC (ccRCCs) and chromophobe RCC [2]. Once sarcomatoid features have been detected in a tumor sample, the tumor is classified as a sarcomatoid RCC (sRCC). The prognosis of metastatic sRCC is poor, with a median overall survival (OS) in the majority of cases being less than one year [3,4,5]. Notably, the percentage of cells with sarcomatoid features determines the prognosis, and a higher proportion is associated with an increased risk of local relapse and inferior OS [2,6]. The International Society of Urologist Pathologists (ISUP) classification recognizes sRCC and rhabdoid RCC as aggressive RCC subsets [7]. sRCC is known to have an especially aggressive behavior, evidenced by a high number of recurrences and death, and, historically, is generally considered to have very limited therapeutic options [8]. Although the data supporting the activity of commonly used drugs such as tyrosine kinase inhibitors (TKIs) and mammalian target of rapamycin (mTOR) inhibitors are either retrospective or extrapolated from trials involving mainly ccRCC and overall very limited in sRCC [9], the systemic therapy recommendations broadly follow treatment approach in ccRCC. Furthermore, the role of CN in sRCC remains somewhat unclear with regard to the rapid evolution of systemic therapy, because of conflicting data and the absence of prospective randomized trials [10]. Moreover, the role of CN has often been disputed in this patient population due to unfavorable outcomes [11]. The question of whether to perform CN in all mRCC patients remains one of the most pertinent issues affecting to a great extent patient outcomes.

The administration of immune checkpoint inhibitors (ICI) has become an accepted standard of care for patients with many tumor types [12]. Subgroup analyses of sRCC in the first line setting in recent phase 3 clinical trials confirmed the efficacy of ICI-based regimens compared to sunitinib [13,14,15,16]. In a post hoc analysis of the phase III CheckMate 214 trial, anti-PD-1 therapy with nivolumab plus anti-CTLA4 therapy with ipilimumab showed an impressive overall response rate (ORR) and OS benefit in comparison with sunitinib in treatment-naive sRCC patients with intermediate and poor-risk prognosis [13,17]. Notably, the complete response rate (CR) in nivolumab-plus-ipilimumab-treated sRCC patients reached 18.9% versus 3.1% for the sunitinib arm, demonstrating a tremendous benefit for so far hopeless patients [13]. Indeed, with ORR over 50%, CR of 18.9%, and median OS (mOS) exceeding two years, the combination of nivolumab plus ipilimumab has established a new therapeutic standard for sRCC patients. Other active regimens for sRCC include the combination of pembrolizumab plus axitinib in the KEYNOTE-426 trial, with a subset analysis showing ORR of 58.8%, CR of 11.8%, 12-month OS of 83.4% [14], and the avelumab plus axitinib regimen in the JAVELIN Renal 101 trial, with ORR of 47%, CR of 4%, and 12-month OS of 83% [18]. Moreover, atezolizumab plus bevacizumab in the subset analysis of the IMmotion 151 trial showed significant benefit in all parameters regardless of the prognostic group, including response rate of 49%, CR of 10%, and 12-month OS of 69% [15]. Finally, based on a meta-analysis published by Iacovelli et al., ICI-based combinations showed an improved outcome in all parameters, including progression-free survival (PFS) and OS, in comparison with sunitinib (until recently considered a standard of care), with a decline of over 40% of progression (HR = 0.56; *p* < 0.0001) and mortality (HR = 0.56; *p* = 0.001) risks [19]. It should be noted that the sarcomatoid proportion within the tumor (<10%, >50%, or pure sarcomatoid histology) was not addressed in the results of effectivity in these trials.

We reviewed our database from 2015 until 2021 and identified six patients with synchronous mRCC with sarcomatoid histology who received a combination of nivolumab with ipilimumab as front-line therapy. Herein, we report the six cases, focusing on the timing of cytoreductive nephrectomy that was considered upfront or delayed depending on patient response to immunotherapy.

## 2. Case Presentation

The clinical characteristics of the patients included in this case series are described here, and important data are summarized in Table 1.

### 2.1. Case 1

**Figure 1:**
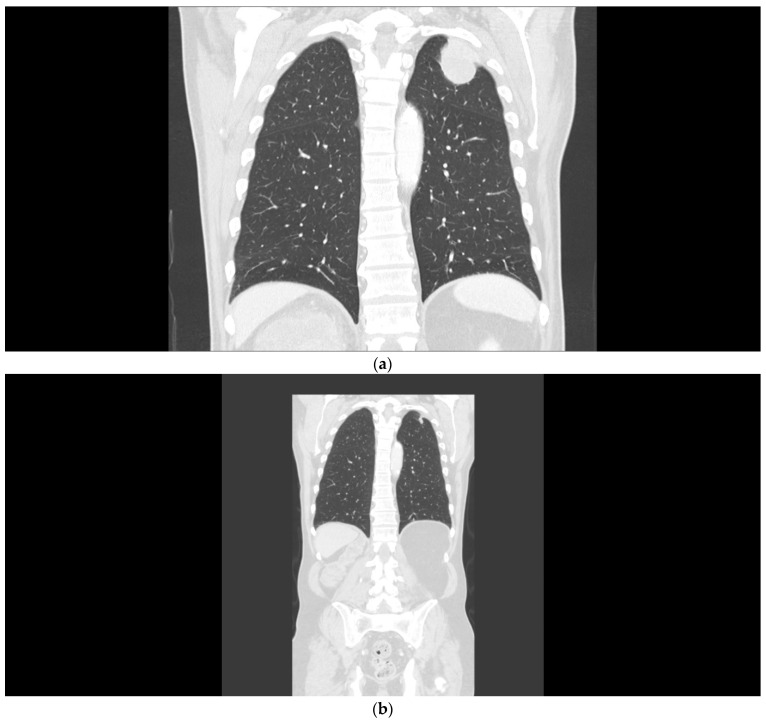
(**a**) Patient 1, pretreatment CT of the chest demonstrating lung metastases. (**b**) Patient 1, marked tumor reduction after the combination regimen of ipilimumab with nivolumab.

A 68-year-old man presented in March 2020 with a large tumor of the right kidney with synchronous metastases to the lungs, mediastinum, left adrenal gland, and extensive retroperitoneal lymphadenopathy (Figure 1a). The medical history was otherwise unremarkable. The patient underwent cytoreductive nephrectomy regardless of the presence of poor prognostic features according to the IMDC, including anemia, hypercalcemia, thrombocytosis, interval to therapy initiation, and Karnofsky performance status (KPS) 2, in April 2020. Histological examination confirmed a renal cell carcinoma with sarcomatoid and rhabdoid features. In May 2020, the patient received four cycles of nivolumab (3 mg/kg) plus ipilimumab (1 mg/kg) given every three weeks. Within a few weeks of therapy, the patient reported significant relief. Lab tests, including blood count and serum calcium, normalized, and KPS improved to 0. A follow-up CT scan showed partial response in all evaluated lesions (Figure 1b). The only adverse event was itching, that did not require any intervention. The patient continued with nivolumab monotherapy at a 480 mg flat dose until April 2021, when the therapy was interrupted due to Common Terminology Criteria for Adverse Events (CTCAE) grade 3 dermatitis. Systemic prednisone was initiated at 0.5 mg/kg, and the rash decreased within a few weeks. Upon improvement, the therapy with nivolumab was reintroduced. After 25 months, in June 2022, the patient remains on therapy, with a minimal residual tumor on CT scan and no additional adverse events.

### 2.2. Case 2

**Figure 2:**
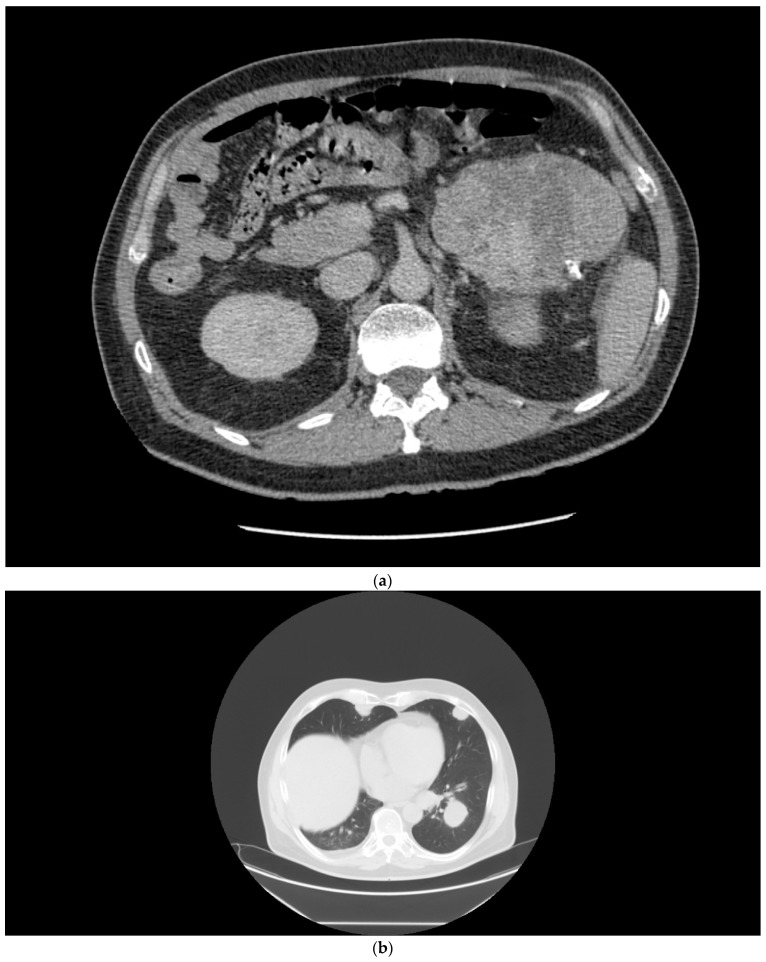

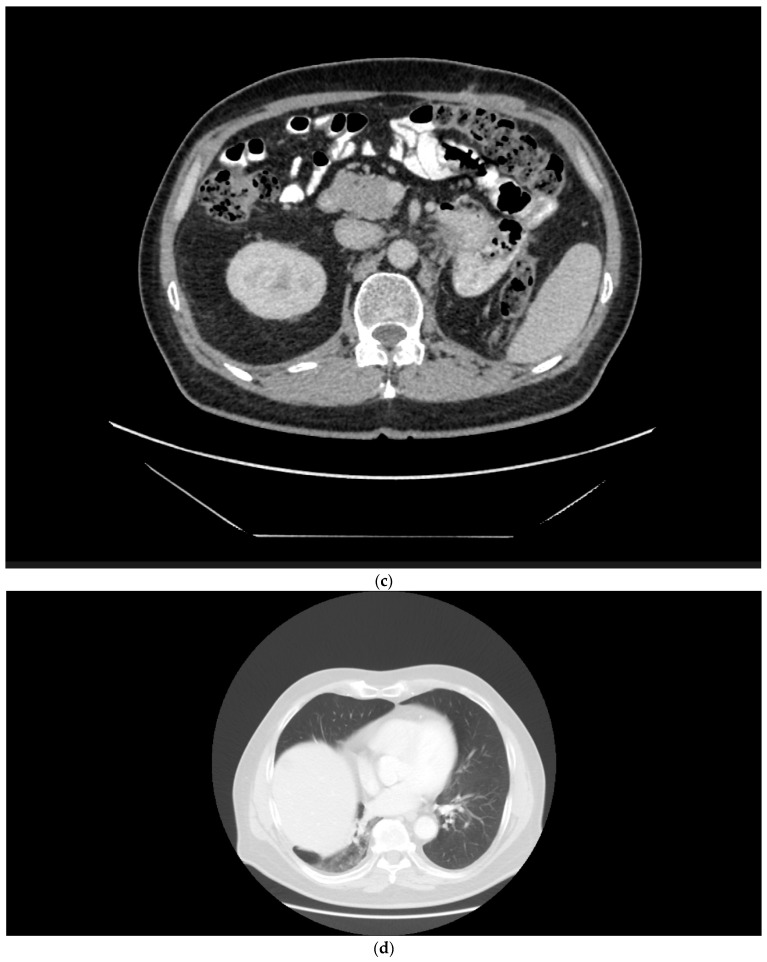
(**a**) Patient 2, pretreatment CT of the renal tumor. (**b**) Patient 2, pretreatment CT of the chest demonstrating lung metastases. (**c**) Patient 2, a large mass in the retroperitoneum. (**d**) Patient 2, major partial response after the combination regimen of ipilimumab with nivolumab.

A 58-year-old male patient presented in November 2014 with a left renal mass and bulky retroperitoneal lymphadenopathy (Figure 2a). The patient had a short history of arterial hypertension and sinus tachycardia. Laboratory examinations at the time of diagnosis showed anemia, thrombocytosis, neutrophilia, and alanine aminotransferase (ALT) and aspartate aminotransferase (AST) CTCAE grade 1 elevation. The patient had the tumor removed in November 2014, but the surgery was only cytoreductive and not radical, leaving a left residual mass in the retroperitoneum. Histological examination revealed sRCC pT3pN1M0. A CT scan two months after surgery showed multiple lung metastases, 3 cm in size (Figure 2b), and a large mass in the retroperitoneum (Figure 2c). The patient had a poor prognosis score according to IMDC (anemia, neutrophilia, thrombocytosis, KPS, and interval to therapy initiation). The laboratory parameters of liver function improved after surgery and were within normal range. In March 2015, the patient was enrolled in the CheckMate 214 trial and received the combination therapy of nivolumab plus ipilimumab at a standard dose for four cycles, then continued with nivolumab monotherapy (3 mg/kg) every two weeks, achieving major partial response on CT examination within two months (Figure 2d). Soon after therapy initiation, the patient felt relieved, and his blood count was normalized. The patient continued with the therapy until October 2021, when the therapy was permanently stopped due to grade 3 ALT and AST elevation. Intravenous methylprednisone 1 mg/kg was immediately initiated. Liver tests gradually returned to normal, and all clinical symptoms accompanying the course of therapy that the patient did not consider, including grade 1 diarrhea, grade 2 insomnia, and grade 1 joint pain, disappeared. The patient continues to be followed. At the last visit in May 2022, he was without evidence of disease activity.

### 2.3. Case 3

A 53-year-old man presented in March 2020 with nausea and vomiting. Gastroscopy detected a duodenal ulcer that was subsequently treated with proton pump inhibitors, while an abdominal ultrasound showed an incidental tumor mass in the left kidney. A CT scan confirmed a large heterogenous tumor of the left kidney and revealed multiple small lung metastases. The patient was scheduled for CN to be followed by systemic immunotherapy. In June 2020, the tumor was surgically removed, with negative margins, and histology confirmed it as sRCC. Given the IMDC poor-risk score (anemia, neutrophilia, and interval to therapy initiation), combination immunotherapy was initiated in July 2020. The therapy was stopped for meningoencephalitis caused by Lyme disease a week after therapy initiation. Two weeks after recovery, the patient had to be rehospitalized for grade 3 ALT, grade 4 AST elevation, and grade 3 hyperbilirubinemia. Rapid improvement of liver tests after intravenous methylprednisone (2 mg/kg) confirmed immune-related adverse events (irAE). A follow-up CT scan showed partial response of the lung metastasis and a large cystic lesion in the original tumor site suspicious of disease recurrence. The patient remained asymptomatic but refused to undergo any therapy at that time. After confirmation of disease progression in April 2021, therapy with cabozantinib was started, which, however, was poorly tolerated, and early progression was evident on CT examination in August 2021. The patient was then unsuccessfully screened (due to only one preceding cycle of ICI therapy, with a minimum of two cycles required) for the clinical trial with belzutifan. Consequently, a nivolumab 240 mg flat dose was reintroduced in December 2021, leading to stable disease and no further adverse events. At the last visit in July 2022, the patient had stable disease on CT examination.

### 2.4. Case 4

A 70-year-old male patient presented in December 2019 with significant weight loss, anorexia, and pain in the lumbar spine. The patient had a history of arterial hypertension and a pacemaker for a complete heart block and had undergone a recent surgery for total endoprosthesis of the left hip (in December 2019). A CT examination showed a right kidney tumor and a thrombus in vena cava inferior (VCI) with bilateral retroperitoneal and mediastinal lymphadenopathy, stage cT3bN1M1. Laboratory examination at the time of diagnosis showed anemia, neutrophilia, thrombocytosis, and coagulopathy. Considering the poor prognostic score as well as coagulopathy, it was decided to perform a biopsy and administer a systemic therapy, followed by deferred CN. The renal tumor biopsy confirmed an sRCC, and the patient received nivolumab plus ipilimumab in July 2020. KPS was 70% on the day of therapy initiation. The patient died ten days later, presumably due to rapid disease progression.

### 2.5. Case 5

A 71-year-old female patient presented in January 2019 with a significant weight loss. A CT scan showed a tumor in the right kidney, 75 mm large in size, a metastasis in the right adrenal gland, and several small nodules in the lungs and pleura, suspicious of metastases. The patient had a history of diabetes mellitus, arterial hypertension, chronic obstructive lung disease, gastric ulcer, and glaucoma. Laboratory tests were within normal ranges, categorizing the patient as having an intermediate prognosis. The CN was performed in March 2019, resulting in the removal of the affected kidney and of the metastasis in the adrenal gland. Histological examination revealed sRCC stage pT3N0M1. The subsequent CT in June 2019 showed a solitary metastatic lesion in the left adrenal gland and stable nodules in the lungs. The therapy with nivolumab plus ipilimumab was initiated in August 2019. Two months later, a control CT scan demonstrated progression of the metastatic lesion in the adrenal gland, but this finding could not be verified on the confirmatory CT scan in January 2020. The patient continued with the immunotherapy, completing four cycles of combination treatment followed by nivolumab monotherapy until disease progression manifested by clinical deterioration and an increase in the size of the metastasis in the left adrenal gland in March 2020. At the end of March 2020, therapy with sunitinib was initiated, which was interrupted after the first cycle due to grade 2 thrombocytopenia. The dose of sunitinib was decreased to 37.5 mg/day, which was well tolerated. Sunitinib administration was terminated in September 2020 for disease progression (enlargement of the left adrenal gland), headache, and overall worsening. In May 2022, the patient was still alive, 18 months after the last CT examination, with a stable overall condition and without any further systemic therapy.

### 2.6. Case 6

A 66-year-old male patient with a history of recurrent urolithiasis, diabetes mellitus on insulin therapy, and stroke (with no permanent disability) presented with lumbar pain in October 2017. A CT examination revealed a large expansion in the retroperitoneum (93 × 67 × 112 mm) in close proximity to the right kidney, several enlarged retroperitoneal lymph nodes, two lesions in the right adrenal gland, and a small lesion (24 mm × 18 mm) in the right kidney. Given the intermediate risk score (thrombocytosis and interval to therapy initiation) according to IMDC and KPS 90%, the patient underwent radical right nephrectomy in October 2017, accompanied by the removal of the large metastasis in the retroperitoneum, with surgical stage being pT3apN1pM1, and histological classification as sRCC. Subsequently, the patient participated in the WO39210 trial, receiving only a placebo, not atezolizumab, as ascertained in March 2020, when disease progression with a growing lymph node in the retroperitoneum was detected on CT. The systemic therapy with nivolumab plus ipilimumab was initiated in April 2020. The patient received three cycles of ICIs, and the therapy had to be terminated in June 2020 due to grade 4 colitis with concurrent acute renal failure (urea 39 mmol/L and creatinine 265 µmol/L) and thyrotoxicosis. Therapy with methylprednisolone 1mg/kg was immediately started, and the dose was increased to 2 mg/kg three days later because of no response. A coloscopic biopsy confirmed colitis. Since colitis was steroid-refractory, infliximab (5 mg/kg) was administered, resulting in prompt relief within ten days after symptoms initiation. Despite the very slow titration of steroids lasting over a year, the patient had to continue the therapy (prednisone 5 mg/day) permanently due to repeated diarrhea recurrence. The patient died, probably due to disease progression, in June 2022.

## 3. Discussion

Here, we presented six cases of mRCC patients with sarcomatoid histology treated with the combination of nivolumab plus ipilimumab in the front-line setting. Five of the patients underwent upfront CN prior to systemic therapy, which resulted in durable responses. Considering the adverse histological subtype and poor prognosis of these patients, an OS exceeding 24 months is far beyond what would be expected. Moreover, two of the patients featuring poor prognostic characteristics and KPS of 70% at the beginning of therapy experienced a prompt symptomatic improvement and durable major partial response to ICIs following CN that would be unlikely prior to the advent of ICI. To the best of our knowledge, this may be the first report of its kind in the literature.

ICIs represent a major advancement in the therapeutic approach aiming at cancer cell elimination. ICIs are particularly effective in tumors with a high burden of immunogenic antigens, such as melanoma and non-small cell lung carcinoma (NSCLC), and present a great potential to control these tumor types. As our understanding of the immune profile of sRCC evolves, the findings that programmed cell death ligand-1 (PD-L1) and tumor-infiltrating lymphocytes (TILs) are frequently expressed in sRCC underpin the activity of ICIs and their use to treat this tumor type, as supported by successful clinical trials’ results. Given the activity of ICI-based regimens in sRCC reported in clinical trials [8,13,17], the incorporation of ICI, aimed to stimulate the immune response against tumors by targeting the regulatory checkpoints PD-1/PD-L1 and/or CTLA-4, as a standard of sRCC therapy is indisputable. On the other hand, the mechanism for this enhanced response to immunotherapy is unclear but may relate to the disease biology. Next-generation sequencing revealed different driver mutations in sRCC compared to ccRCC tumors [20]. The sarcomatoid as well as the epithelial parts of sRCC contain identical genomic features harboring high genomic instability, p53 mutations, lack of von Hippel–Lindau (VHL) gene mutations, high Ki-67, and increased Teff signature [20,21]. One of the mechanisms of the enhanced responsiveness could be a higher immunogenic profile and PD-L1 expression in sRCC, since higher PD-L1 expression and increased CD8-positive cell density have been observed in sRCC compared to non-sarcomatoid RCC tumors [8,15]. Moreover, PD-L1 expression has a prognostic implication, as demonstrated in prior studies [22,23]. Finally, higher PD-L1 expression is associated with a higher grade within a tumor [24]. However, Gupta et al. did not show a prognostic impact of PD-L1 expression in a cohort of patients with high-grade tumors [24]. Besides, these observations have the obvious limitation of tumor heterogeneity. The need to identify biomarkers of response is obvious. For example, 9p24.1 amplification, leading to constitutive expression of PD-L1, appeared to represent an encouraging predictor of response to immunotherapy in RCC [24]. Meanwhile, Yoshida et al. published data on three different histological phenotypes, i.e., eosinophilic, mixed, and clear-type ccRCC, underlined by different mechanisms, that significantly correlate with prognosis and show distinct responses to angiogenic or immune-mediated blockade [25]. Another critical factor is the tumor microenvironment (TME), characterized by an inflamed stroma with extensive immune infiltrates in sRCC [26]. Interestingly, Wang et al. described a correlation of inflammation in the TME with anemia and thrombocytosis, which are two out of six IMDC risk factors, suggesting a systemic immune reaction derived from the tumor [26]. Finally, the genetic heterogeneity of sRCC is reflected in the variability of the tumor response to ICI. Meanwhile, several molecular drivers of ICI resistance have been identified, including enrichment of transforming growth factor beta (TGF-beta) signaling [27].

sRCC is typically characterized by a large primary tumor or bulky disease at initial presentation, and up to 80% of patients have metastatic disease at the time of presentation [10]. The role of CN in sRCC remains a matter of debate. In general, defining the role of CN in mRCC represents not only a clinical challenge [28,29] but often a pivotal moment in determining a patient outcome. The high activity of ICI in patients with sarcomatoid histology underscores the need to re-evaluate CN role in the initial management of patients with this rare disease. Based on the experience with the present cohort, we speculate that removing the large primary tumor could have had an impact on the response to subsequent immunotherapy. This hypothesis is in concordance with the European Society for Medical Oncology (ESMO) guidelines recommending CN in the presence of large primary tumors [30]. Performing CN in five of the patients in the present series was feasible and facilitated the response to subsequent systemic therapy, possibly due to tumor debulking. Furthermore, two of the patients with initially poor prognosis had a prompt and very favorable responses, with major partial responses on CT examination. On the other hand, we do not know whether immunotherapy could have had the same effect without previous CN, since a reduction of the tumor mass might have not been necessary, and the tumor would have decreased in size anyway following immunotherapy.

In the era of targeted therapy, CN was associated with improved OS in several large retrospective observational studies [31,32,33,34], and its benefit was also demonstrated in mRCC of non-clear cell histology [35]. Sarcomatoid differentiation portends a worse prognosis, regardless of a clear cell or non-clear cell origin [36]. Nevertheless, an improved outcome was also noted in patients with sRCC undergoing CN versus no surgery [10,33]. These results advocate for CN in this patient population [37]. However, data on the role of CN in patients with non-clear cell histology are scarce and only retrospective. Considering the optimal approach in a patient with mRCC of non-clear cell histology, we should adopt an individualized strategy and balance the pros and cons with regard to the benefit of CN versus delayed initiation of systemic therapy. Adashek et al. published data on patients with sarcomatoid dedifferentiation undergoing CN showing poor prognosis and limited or no benefit from the surgery [38]. The authors conclude that by performing a pretreatment biopsy with detection of unfavorable histology such as a sarcomatoid histology, the treatment should be switched to systemic therapy conferring meaningful strategic benefit to the patient [38]. A retrospective study (391 patients, 5.6% sRCC) reported by Singla et al. demonstrated a survival benefit in patients treated with CN plus ICIs compared to patients receiving ICIs alone, from a registry-based cohort of patients [39]. Meanwhile, patients with administered a preoperative setting of ICIs showed a better outcome in terms of grade and tumor stage compared to patients receiving CN upfront. Moreover, pathological CR (pCR) in the primary tumor was identified in 10% of patients receiving ICIs preoperatively [39]. However, it should be kept in mind that patients with upfront CN had more favorable tumor characteristics. On multivariable analysis, no predictors of favorable outcomes regarding CN timing were identified [39].

Most importantly, CN is not recommended for patients with poor prognostic features based on several retrospective studies [32,33,40] as well as the prospective CARMENA trial results [41], and this fact is reflected in international guidelines such as the European Association of Urology (EAU) and ESMO guidelines [30,42]. Data from the SURTIME trial support immediate (upfront) CN only for a subset of patients with mRCC [43]. In the present series, three of the patients were in the poor prognostic category but still showed a durable treatment response. Based on this experience, we do not think that a poor prognosis should be considered an absolute contraindication when deciding whether to perform an upfront CN. It should be noted that both CARMENA and SURTIME prospective trials challenge the role of CN as such. It is obvious that selecting the patients who benefit most from CN is of utmost importance. Recruitment into prospective trials of CN was rather difficult. Thus, there is little doubt that in rare conditions such as sRCC, no prospective trial dealing with the role of CN will ever be pursued.

A post hoc analysis of the SURTIME trial showed an advantage of deferred CN with regard to improved management of systemic therapy because of earlier administration of sunitinib as well as longer duration of therapy leading to better disease control [44]. In the cohort with immediate CN, 19.6% of the patients reported disease progression 4 weeks following surgery, with median time to systemic therapy initiation of 39.5 days and 46% of the patients showing disease progression 4 months from CN, in contrast with only 32.7% in the deferred arm [44]. From this point of view, it seems more convenient to start with systemic therapy to achieve disease control; on the other hand, the window of opportunity for removing the primary tumor can be missed irreversibly. Interestingly, a retrospective analysis of 71 mRCC patients treated with ipilimumab and nivolumab upfront with the option of deferred CN in responding patients showed a 33.3 % response rate in the primary site and a 91.3% overall response rate at metastatic sites regardless of the prognostic group [45]. CN was performed in 18.8% of the patients after a median time of 13 months [45].

Recently, data from a cohort study from the National Cancer Database published by Chakiryan et al. did not demonstrate a survival benefit for CN in patients with mRCC using instrumental variable analysis [46]. The authors claim that the indication for CN is primarily driven by factors that are not reflected in observational studies, leading to confounding results. Distance from patient’s residence influencing the decision of CN could by one of these factors. It should also be noted that there are potential complications arising from CN, which may jeopardize further systemic therapy. In terms of wound healing after an extensive surgical procedure, the patient could be exposed to deterioration of immunosurveillance and rapid disease progression. Delay of systemic therapy is one of the major concerns regarding CN. On the other hand, immunotherapy itself may not influence wound healing, but fibrotic changes following successful immunotherapy do occur, making the surgical procedure a challenge. The morbidity of the surgical procedure should not be neglected [47,48]. Currently undergoing prospective trials addressing the role of CN in combination with ICIs in mRCC, including the PROBE trial and the NORDIC-SUN, are enrolling patients with all histological subtypes; nevertheless, the number of sRCC patients included will probably be limited [49]. Therefore, we can learn from retrospective data and case reports about this patient population [35,50,51] and perhaps joint multi-institutional efforts to establish a concept of treatment recommendations.

## 4. Conclusions

Tailoring the treatment strategy for an aggressive disease such as sRCC remains a challenge. Undoubtedly, immunotherapy represents a current gold standard of systemic therapy in sRCC. Due to the paucity of prospective data, defining the role of CN in the immunotherapy era continues to be a matter of debate. The present findings underscore the need for an individualized approach when considering a multimodality treatment to weigh the potential benefit of primary tumor mass reduction against the risk of perioperative complications and systemic therapy delay. Although this concept may warrant prospective validation, this may be difficult for rare RCC subtypes such as tumors with sarcomatoid features. The optimal management of rare types of mRCC, including sRCC, remains an unmet medical need but, in the foreseeable future, it will be guided by retrospective case series like the present cohort.

## Figures and Tables

**Table 1:** Patient Characteristics.

Patient	Age (Years)	Sex (Male/ Female)	IMDC Score	Initial Stage	Size of the Primary Tumor (mm)	% Sarcomatoid Component	Number of Metastatic Sites	Treatment Duration (Days)	Time from CN to ICI Initiation (Days)	Survival from Initial Diagnosis (Months)/Latest Control	Toxicity
1	68	M	5	T2bN1M1	100	100	3	720+	22	26+/(June 2022)	dermatitis
2	58	M	5	T3aN1M0	70	100	2	2419	99	90+/(May 2022)	hepatotoxicity
3	53	M	3	T3aN1M1	90	80	1	21	36	25+/(July 2022)	hepatotoxicity
4	70	M	6	T3cN1M1	120	NE	1	10	NA	0.3	none
5	71	F	1	T3aN0M1	75	75	1	188	144	35+/(May 2022)	none
6	66	M	1	T3aN1M1	42	95	1	42	913	56	colitis

NA not applicable, NE not evaluable

## Data Availability

The authors declare that all data concerning this case series are provided within the manuscript.

## Abbreviations

IMDC: International Metastatic RCC Database Consortium; RCC: renal carcinoma; ccRCC: clear cell RCC; sRCC: sarcomatoid RCC; OS: overall survival; ISUP: International Society of Urologist Pathologists; CN: cytoreductive nephrectomy; TKIs: tyrosine kinase inhibitors; mTOR: mammalian target of rapamycin; ICIs: immune checkpoint inhibitors; ORR: objective response rate; TILs: tumor infiltrating lymphocytes; CR: complete response rate; PFS: progression-free survival; KPS: Karnofsky performance status; CTCAE: Common Terminology Criteria for Adverse Events; ALT: alanine aminotransferase; AST: aspartate aminotransferase; irAE: immune-related adverse event; NSCLC: non-small cell lung cancer; PD-L1: programmed cell death ligand-1; VHL: Von Hippel-Lindau; TME: tumor microenvironment; TGF-beta: transforming growth factor beta; pCR;pathologic complete response; ESMO: European Society for Medical Oncology; EAU: European Association of Urology.

## References

[1] Sung H., Ferlay J., Siegel R.L., Laversanne M., Soerjomataram I., Jemal A., Bray F. (2021). Global Cancer Statistics 2020: GLOBOCAN Estimates of Incidence and Mortality Worldwide for 36 Cancers in 185 Countries. CA Cancer J. Clin..

[2] Kim T., Zargar-Shoshtari K., Dhillon J., Lin H.Y., Yue B., Fishman M., Sverrisson E.F., Spiess P.E., Gupta S., Poch M.A. (2015). Using percentage of sarcomatoid differentiation as a prognostic factor in renal cell carcinoma. Clin. Genitourin. Cancer.

[3] de Velasco G., McKay R.R., Lin X., Moreira R.B., Simantov R., Choueiri T.K. (2017). Comprehensive Analysis of Survival Outcomes in Non-Clear Cell Renal Cell Carcinoma Patients Treated in Clinical Trials. Clin. Genitourin. Cancer.

[4] Zhang B.Y., Thompson R.H., Lohse C.M., Leibovich B.C., Boorjian S.A., Cheville J.C., Costello B.A. (2015). A novel prognostic model for patients with sarcomatoid renal cell carcinoma. BJU Int..

[5] Kyriakopoulos C.E., Chittoria N., Choueiri T.K., Kroeger N., Lee J.L., Srinivas S., Knox J.J., Bjarnason G.A., Ernst S.D., Wood L.A. (2015). Outcome of patients with metastatic sarcomatoid renal cell carcinoma: Results from the International Metastatic Renal Cell Carcinoma Database Consortium. Clin. Genitourin. Cancer.

[6] Adibi M., Thomas A.Z., Borregales L.D., Merrill M.M., Slack R.S., Chen H.C., Sircar K., Murugan P., Tamboli P., Jonasch E. (2015). Percentage of sarcomatoid component as a prognostic indicator for survival in renal cell carcinoma with sarcomatoid dedifferentiation. Urol. Oncol..

[7] Moch H., Cubilla A.L., Humphrey P.A., Reuter V.E., Ulbright T.M. (2016). The 2016 WHO Classification of Tumours of the Urinary System and Male Genital Organs-Part A: Renal, Penile, and Testicular Tumours. Eur. Urol..

[8] Debien V., Thouvenin J., Lindner V., Barthélémy P., Lang H., Flippot R., Malouf G.G. (2019). Sarcomatoid Dedifferentiation in Renal Cell Carcinoma: From Novel Molecular Insights to New Clinical Opportunities. Cancers.

[9] Voss M.H., Bastos D.A., Karlo C.A., Ajeti A., Hakimi A.A., Feldman D.R., Hsieh J.J., Molina A.M., Patil S., Motzer R.J. (2014). Treatment outcome with mTOR inhibitors for metastatic renal cell carcinoma with nonclear and sarcomatoid histologies. Ann. Oncol..

[10] Alevizakos M., Gaitanidis A., Nasioudis D., Msaouel P., Appleman L.J. (2019). Sarcomatoid Renal Cell Carcinoma: Population-Based Study of 879 Patients. Clin. Genitourin. Cancer.

[11] Shuch B., Said J., La Rochelle J.C., Zhou Y., Li G., Klatte T., Kabbinaavar F.F., Pantuck A.J., Belldegrun A.S. (2009). Cytoreductive nephrectomy for kidney cancer with sarcomatoid histology--is up-front resection indicated and, if not, is it avoidable?. J. Urol..

[12] von Minckwitz G., Untch M., Blohmer J.U., Costa S.D., Eidtmann H., Fasching P.A., Gerber B., Eiermann W., Hilfrich J., Huober J. (2012). Definition and impact of pathologic complete response on prognosis after neoadjuvant chemotherapy in various intrinsic breast cancer subtypes. J. Clin. Oncol..

[13] Tannir N.M., Signoretti S., Choueiri T.K., McDermott D.F., Motzer R.J., Flaifel A., Pignon J.C., Ficial M., Frontera O.A., George S. (2021). Efficacy and Safety of Nivolumab Plus Ipilimumab versus Sunitinib in First-line Treatment of Patients with Advanced Sarcomatoid Renal Cell Carcinoma. Clin. Cancer Res..

[14] Rini B.I., Plimack E.R., Stus V., Gafanov R., Hawkins R., Nosov D., Pouliot F., Soulieres D., Melichar B., Vynnychenko I. (2019). Pembrolizumab (pembro) plus axitinib (axi) versus sunitinib as first-line therapy for metastatic renal cell carcinoma (mRCC): Outcomes in the combined IMDC intermediate/poor risk and sarcomatoid subgroups of the phase 3 KEYNOTE-426 study. J. Clin. Oncol..

[15] Rini B.I., Motzer R.J., Powles T., McDermott D.F., Escudier B., Donskov F., Hawkins R.E., Bracarda S., Bedke J., De Giorgi U. (2019). Atezolizumab (atezo) plus bevacizumab (bev) versus sunitinib (sun) in pts with untreated metastatic renal cell carcinoma (mRCC) and sarcomatoid (sarc) histology: IMmotion151 subgroup analysis. J. Clin. Oncol..

[16] Choueiri T.K., Larkin J., Pal S., Motzer R.J., Rini B.I., Venugopal B., Alekseev B., Miyake H., Gravis G., Bilen M.A. (2021). Efficacy and correlative analyses of avelumab plus axitinib versus sunitinib in sarcomatoid renal cell carcinoma: Post hoc analysis of a randomized clinical trial. ESMO Open.

[17] Hwang J.K., Agarwal N., Brugarolas J., Zhang T. (2021). Checking the Hippo in Sarcomatoid Renal Cell Carcinoma with Immunotherapy. Clin. Cancer Res..

[18] Choueiri T.K., Larkin J.M.G., Pal S.K., Motzer R.J., Venugopal B., Alekseev B.Y., Miyake H., Gravis G., Bilen M.A., Chudnovsky A. (2019). Efficacy and biomarker analysis of patients (pts) with advanced renal cell carcinoma (aRCC) with sarcomatoid histology (sRCC): Subgroup analysis from the phase III JAVELIN renal 101 trial of first-line avelumab plus axitinib (A plus Ax) vs sunitinib (S). Ann. Oncol..

[19] Iacovelli R., Ciccarese C., Bria E., Bracarda S., Porta C., Procopio G., Tortora G. (2020). Patients with sarcomatoid renal cell carcinoma—Re-defining the first-line of treatment: A meta-analysis of randomised clinical trials with immune checkpoint inhibitors. Eur. J. Cancer.

[20] Malouf G.G., Ali S.M., Wang K., Balasubramanian S., Ross J.S., Miller V.A., Stephens P.J., Khayat D., Pal S.K., Su X. (2016). Genomic Characterization of Renal Cell Carcinoma with Sarcomatoid Dedifferentiation Pinpoints Recurrent Genomic Alterations. Eur. Urol..

[21] Turajlic S., Xu H., Litchfield K., Rowan A., Horswell S., Chambers T., O’Brien T., Lopez J.I., Watkins T.B.K., Nicol D. (2018). Deterministic Evolutionary Trajectories Influence Primary Tumor Growth: TRACERx Renal. Cell.

[22] Kawakami F., Sircar K., Rodriguez-Canales J., Fellman B.M., Urbauer D.L., Tamboli P., Tannir N.M., Jonasch E., Wistuba I.I., Wood C.G. (2017). Programmed cell death ligand 1 and tumor-infiltrating lymphocyte status in patients with renal cell carcinoma and sarcomatoid dedifferentiation. Cancer.

[23] Joseph R.W., Millis S.Z., Carballido E.M., Bryant D., Gatalica Z., Reddy S., Bryce A.H., Vogelzang N.J., Stanton M.L., Castle E.P. (2015). PD-1 and PD-L1 Expression in Renal Cell Carcinoma with Sarcomatoid Differentiation. Cancer Immunol. Res..

[24] Gupta S., Cheville J.C., Jungbluth A.A., Zhang Y., Zhang L., Chen Y.B., Tickoo S.K., Fine S.W., Gopalan A., Al-Ahmadie H.A. (2019). JAK2/PD-L1/PD-L2 (9p24.1) amplifications in renal cell carcinomas with sarcomatoid transformation: Implications for clinical management. Mod. Pathol..

[25] Yoshida T., Ohe C., Ikeda J., Atsumi N., Ohsugi H., Sugi M., Higasa K., Saito R., Tsuta K., Matsuda T. (2021). Eosinophilic features in clear cell renal cell carcinoma correlate with outcomes of immune checkpoint and angiogenesis blockade. J. Immunother. Cancer.

[26] Wang T., Lu R., Kapur P., Jaiswal B.S., Hannan R., Zhang Z., Pedrosa I., Luke J.J., Zhang H., Goldstein L.D. (2018). An Empirical Approach Leveraging Tumorgrafts to Dissect the Tumor Microenvironment in Renal Cell Carcinoma Identifies Missing Link to Prognostic Inflammatory Factors. Cancer Discov..

[27] Wang Z., Kim T.B., Peng B., Karam J., Creighton C., Joon A., Kawakami F., Trevisan P., Jonasch E., Chow C.W. (2017). Sarcomatoid Renal Cell Carcinoma Has a Distinct Molecular Pathogenesis, Driver Mutation Profile, and Transcriptional Landscape. Clin. Cancer Res..

[28] Singla N., Ghandour R.A., Margulis V. (2019). Is cytoreductive nephrectomy relevant in the immunotherapy era?. Curr. Opin. Urol..

[29] Singla N., Hakimi A.A., Margulis V. (2019). Editorial: The evolving role of cytoreductive nephrectomy. Curr. Opin. Urol..

[30] Escudier B., Porta C., Schmidinger M., Rioux-Leclercq N., Bex A., Khoo V., Grünwald V., Gillessen S., Horwich A. (2019). Renal Cell Carcinoma: ESMO Clinical Practice Guidelines for diagnosis, treatment and follow-up. Ann. Oncol..

[31] Conti S.L., Thomas I.C., Hagedorn J.C., Chung B.I., Chertow G.M., Wagner T.H., Brooks J.D., Srinivas S., Leppert J.T. (2014). Utilization of cytoreductive nephrectomy and patient survival in the targeted therapy era. Int. J. Cancer.

[32] Mathieu R., Pignot G., Ingles A., Crepel M., Bigot P., Bernhard J.C., Joly F., Guy L., Ravaud A., Azzouzi A.R. (2015). Nephrectomy improves overall survival in patients with metastatic renal cell carcinoma in cases of favorable MSKCC or ECOG prognostic features. Urol. Oncol..

[33] Heng D.Y., Wells J.C., Rini B.I., Beuselinck B., Lee J.L., Knox J.J., Bjarnason G.A., Pal S.K., Kollmannsberger C.K., Yuasa T. (2014). Cytoreductive nephrectomy in patients with synchronous metastases from renal cell carcinoma: Results from the International Metastatic Renal Cell Carcinoma Database Consortium. Eur. Urol..

[34] Choueiri T.K., Xie W., Kollmannsberger C., North S., Knox J.J., Lampard J.G., McDermott D.F., Rini B.I., Heng D.Y.C. (2011). The Impact of Cytoreductive Nephrectomy on Survival of Patients with Metastatic Renal Cell Carcinoma Receiving Vascular Endothelial Growth Factor Targeted Therapy. J. Urol..

[35] Alhalabi O., Karam J.A., Tannir N.M. (2019). Evolving role of cytoreductive nephrectomy in metastatic renal cell carcinoma of variant histology. Curr. Opin. Urol..

[36] Kassouf W., Sanchez-Ortiz R., Tamboli P., Tannir N., Jonasch E., Merchant M.M., Matin S., Swanson D.A., Wood C.G. (2007). Cytoreductive nephrectomy for metastatic renal cell carcinoma with nonclear cell histology. J. Urol..

[37] Blum K.A., Gupta S., Tickoo S.K., Chan T.A., Russo P., Motzer R.J., Karam J.A., Hakimi A.A. (2020). Sarcomatoid renal cell carcinoma: Biology, natural history and management. Nat. Rev. Urol..

[38] Adashek J.J., Zhang Y., Skelton W.P.t., Bilotta A., Chahoud J., Zemp L., Li J., Dhillon J., Manley B., Spiess P.E. (2020). Dissecting Outcomes: Should Cytoreductive Nephrectomy Be Performed for Patients with Metastatic Renal Cell Carcinoma with Sarcomatoid Dedifferentiation?. Front. Oncol..

[39] Singla N., Hutchinson R.C., Ghandour R.A., Freifeld Y., Fang D., Sagalowsky A.I., Lotan Y., Bagrodia A., Margulis V., Hammers H.J. (2020). Improved survival after cytoreductive nephrectomy for metastatic renal cell carcinoma in the contemporary immunotherapy era: An analysis of the National Cancer Database. Urol. Oncol..

[40] Dilme R.V., Rivas J.G., Campi R., Puente J., Jerez T., Enikeev D., Esperto F., Sierra J.M. (2021). Cytoreductive Nephrectomy in the Management of Metastatic Renal Cell Carcinoma: Is There Still a Debate?. Curr. Urol. Rep..

[41] Mejean A., Ravaud A., Thezenas S., Colas S., Beauval J.B., Bensalah K., Geoffrois L., Thiery-Vuillemin A., Cormier L., Lang H. (2018). Sunitinib Alone or after Nephrectomy in Metastatic Renal-Cell Carcinoma. N. Engl. J. Med..

[42] Bex A., Albiges L., Ljungberg B., Bensalah K., Dabestani S., Giles R.H., Hofmann F., Hora M., Kuczyk M.A., Lam T.B. (2018). Updated European Association of Urology Guidelines for Cytoreductive Nephrectomy in Patients with Synchronous Metastatic Clear-cell Renal Cell Carcinoma. Eur. Urol..

[43] Bex A., Mulders P., Jewett M., Wagstaff J., van Thienen J.V., Blank C.U., van Velthoven R., Del Pilar Laguna M., Wood L., van Melick H.H.E. (2019). Comparison of Immediate vs Deferred Cytoreductive Nephrectomy in Patients with Synchronous Metastatic Renal Cell Carcinoma Receiving Sunitinib: The SURTIME Randomized Clinical Trial. JAMA Oncol..

[44] Abu-Ghanem Y., van Thienen J.V., Blank C., Aarts M.J.B., Jewett M., de Jong I.J., Lattouf J.B., van Melick H.H.E., Wood L., Mulders P. (2022). Cytoreductive nephrectomy and exposure to sunitinib—A post hoc analysis of the Immediate Surgery or Surgery after Sunitinib Malate in Treating Patients with Metastatic Kidney Cancer (SURTIME) trial. BJU Int..

[45] Meerveld-Eggink A., Graafland N., Wilgenhof S., Van Thienen J.V., Lalezari F., Grant M., Szabados B., Abu-Ghanem Y., Kuusk T., Boleti E. (2022). Primary Renal Tumour Response in Patients Treated with Nivolumab and Ipilimumab for Metastatic Renal Cell Carcinoma: Real-world Data Assessment. Eur. Urol. Open. Sci..

[46] Chakiryan N.H., Gore L.R., Reich R.R., Dunn R.L., Jiang D.D., Gillis K.A., Green E., Hajiran A., Hugar L., Zemp L. (2022). Survival Outcomes Associated with Cytoreductive Nephrectomy in Patients with Metastatic Clear Cell Renal Cell Carcinoma. JAMA Netw. Open.

[47] Labbate C., Hatogai K., Werntz R., Stadler W.M., Steinberg G.D., Eggener S., Sweis R.F. (2019). Complete response of renal cell carcinoma vena cava tumor thrombus to neoadjuvant immunotherapy. J. Immunother. Cancer.

[48] Pignot G., Thiery-Vuillemin A., Walz J., Lang H., Bigot P., Werle P., Balssa L., Geoffrois L., Leblanc L., Albigès L. (2020). Nephrectomy after Complete Response to Immune Checkpoint Inhibitors for Metastatic Renal Cell Carcinoma: A New Surgical Challenge?. Eur. Urol..

[49] Kuusk T., Abu-Ghanem Y., Mumtaz F., Powles T., Bex A. (2021). Perioperative therapy in renal cancer in the era of immune checkpoint inhibitor therapy. Curr. Opin. Urol..

[50] Sejima T., Masago T., Yoshida M., Nishi T., Kawabata Y., Tajima Y., Yumioka T., Honda M., Takenaka A. (2021). Pathological eradication of recurrent metastatic renal cell carcinoma with sarcomatoid component by nivolumab plus ipilimumab combination therapy. Int. Cancer Conf. J..

[51] Park J.J., Kellezi O., Hamasha R., Ali A., Alva A.S. (2020). Immunotherapy in metastatic sarcomatoid renal cell carcinoma: A single institution experience. Cancer Treat. Res. Commun..

